# The Permissibility of Biased AI in a Biased World: An Ethical Analysis of AI for Screening and Referrals for Diabetic Retinopathy in Singapore

**DOI:** 10.1007/s41649-024-00315-3

**Published:** 2024-10-31

**Authors:** Kathryn Muyskens, Angela Ballantyne, Julian Savulescu, Harisan Unais Nasir, Anantharaman Muralidharan

**Affiliations:** 1 Centre for Biomedical Ethics, Yong Loo Lin School of Medicine, National University of Singapore, Singapore; 2 Department of Primary Health Care and General Practice, University of Otago, Dunedin, New Zealand; 3 University of Oxford, Oxford, UK

**Keywords:** AI ethics, Bias, Data ethics, Healthcare ethics, Public health ethics, Equity, Utility

## Abstract

A significant and important ethical tension in resource allocation and public health ethics is between utility and equity. We explore this tension between utility and equity in the context of health AI through an examination of a diagnostic AI screening tool for diabetic retinopathy developed by a team of researchers at Duke-NUS in Singapore. While this tool was found to be effective, it was not equally effective across every ethnic group in Singapore, being less effective for the minority Malay population than for the Chinese majority. We discuss the problematic normative nature of bias in health AI and explore the ways in which bias can interact with various forms of social inequalities. From there, we examine the specifics of the diabetic retinopathy case and weigh up specific trade-offs between utility and equity. Ultimately, we conclude that it is ethically permissible to prioritise utility over equity where certain criteria hold. Given that any medical AI is more likely than not to have lingering bias due to bias in the training data that may reflect other social inequalities, we argue that it is permissible to implement an AI tool with residual bias where: (1) its introduction reduces the influence of biases (even if overall inequality is worsened), and/or (2) where the utility gained is significant enough and shared across groups (even if unevenly).

## Introduction

A significant and important ethical tension in resource allocation and public health ethics is between utility and equity. Ideally, we want to introduce and fund health interventions that improve population health and decrease health inequities. However, sometimes these two values are in conflict. When it proves impossible to maximise both utility and equity, we must decide which to prioritise. Furthermore, we must determine when it is ethically acceptable to deploy AI-based tools in a clinical setting that may contribute to health disparities between groups. Thus, there is a need for better guidance on how to pick between imperfect options. In this paper, we will explore a case where the application of a particular form of health AI presents us with one such choice, namely the question of whether implementing a tool that increases wellbeing at the cost of worsening a pre-existing inequity is ethically permissible. We explore this tension between utility and equity in the context of health AI through an examination of a case study of a diagnostic tool for the screening of diabetic retinopathy developed by a team of researchers at Duke-NUS in Singapore. While this tool was found to be effective, it was not equally effective in its detection of diabetic retinopathy across every ethnic group in Singapore, being less effective for the minority Malay population than for the Chinese majority.

In our exploration of the case, we will discuss the problematic normative nature of bias in health AI — a phenomenon that has generated apprehension and diverse strategies to minimise algorithmic bias, but which remains under-theorised from a philosophical or bioethics perspective. There is general agreement that algorithmic bias is a significant concern with respect to AI in health and that such bias should be investigated and minimised where possible. While there are a number of recent articles covering other aspects of AI in clinical application, from AI in support for ethical decision-making (see Biller-Adorno et al. 2022) to epistemological challenges in using AI for diagnostics (see Kempt and Nagel 2022), there has been limited work analysing exactly when bias amounts to an ethical barrier to deploying AI in the clinical context. Specifically, we wish to ask, when does algorithmic bias represent a substantial enough injury to justice that the unfairness would outweigh the potential utility benefits offered by the given AI program, rendering its use ethically unacceptable? The framing of this question admits that some degree of bias or inequality may be ethically tolerable. We postulate that algorithmic bias in AI may be ethically acceptable for two reasons, because (a) systemic bias is already present in the health system in question and (b) the utility gain offered by the AI may ethically outweigh the equity considerations.

For our analysis, we take utility to mean maximising the aggregate health gain for the population. Broadly speaking, utility could be achieved via decreasing morbidity or mortality, improving quality of life, reducing health resource waste, improving triage, improving clinical pathways or processes, or achieving the same health outcome but with reduced costs. According to the World Health Organization (WHO), equity refers to the absence of unfair, avoidable, and remediable differences between groups of people, whether those groups are defined socially, economically, demographically, or geographically or by other dimensions of inequality (e.g. sex, gender, ethnicity, disability, or sexual orientation) (WHO n.d.).

Utility and equity are both important goals of the health system. This has been recognised and articulated in international declarations, such as the Organization for Economic Cooperation and Development (OECD) government policy and the WHO’s public health ethics frameworks and statements. That said, the best ways to achieve and appropriately balance considerations of utility and equity in relation to specific clinical interventions — such as triage scores, resource allocation protocols, clinical decisions aids, or the use of AI apps — is complicated and heavily debated. In part, this is because any given clinical tool is introduced to a real-world health system and social context, which may feature historical injustices, current disparities in care, and unique social and economic power dynamics.

To explore these tensions between utility and equity, we consider a specific case of an AI screening tool for detecting diabetic retinopathy in Singapore. To make our case, we also provide an analysis of several types of bias in AI, and how this interacts with the background injustice present within whatever social context such a tool might be deployed. We explore the various ways in which utility and equity interact in this scenario and attempt to extrapolate insights that can inform ethical decision-making in analogous cases in the future. We hope that our analysis will be illustrative and informative, but we also acknowledge its highly contextual nature, and that different trade-offs may be justifiable within different social contexts and healthcare systems.

## The Case

Diabetes affects a growing number of people worldwide. The International Diabetes Federation (IDF) reports that 10.5% of the adult population (between 20 and 70 years old) has diabetes, estimating that half of those are unaware that they are living with the condition (International Diabetes Federation 2021), and up to 9.5% of the population in Singapore as of 2019 are affected by the disease (Diabetes Singapore n.d.). According to the WHO (2023), “In 2019, diabetes was the direct cause of 1.5 million deaths and 48% of all deaths due to diabetes occurred before the age of 70 years”. It is a chronic and progressive illness that requires careful management in the form of medication, and/or diet and exercise in order to stave off complications. Unmanaged diabetes can lead to the development of many other comorbidities, including effects on the heart, kidney, and other vital organs. Diabetes can also affect the eyes, causing damage to the blood vessels in the retina (diabetic retinopathy) or to the optic nerve (glaucoma) (Mayo Clinic Staff 2023). Early detection and treatment are vital to prevent further complications of these conditions. Diabetic patients also suffer financial burdens, from increased insurance premiums, the cost of medication and treatment, and the risk of job loss in the event that their condition worsens (Lim 2022). For a bit of context, while all Singaporeans and permanent residents receive some insurance coverage for diabetes-related hospitalisation, outpatient treatments, and medication, they are still required to pay at least 15% out of pocket. Moreover, this state insurance is paid for from compulsory medical savings accounts, for those who are able to afford it, but covered by the state for those who are not. Many Singaporeans may also purchase private insurance to improve coverage.

As a chronic and progressive condition, diabetes does not look the same in every patient. Some patients will have much more severe symptoms, and this becomes more likely when the disease is poorly controlled (e.g. where the patient adopts less management strategies, like frequent exercise, careful nutrition, or regular check-ups). To minimise these costs, it is important to screen for and detect the condition and its comorbidities early. To that end, a team from Duke-NUS led by Ting and colleagues (2017) has developed a deep learning system to detect the progression of diabetic retinopathy and glaucoma (hereafter referred to as DLSDR for a deep learning system for diabetic retinopathy). Retinal images in the DLSDR training set were labelled by professional graders, and disagreements were settled by retinal specialists. Where the progression of a disease achieves a certain level, the algorithm tags it as referable and radiologists may use this result to refer the patient to a specialist. While the accuracy of detection by the algorithm was, overall, comparable to human experts, the algorithm exhibited significantly lower sensitivity (97.1%) in detecting referable diabetic retinopathy for Malays than for non-Malays^1^ (99.3% for Indians and 100% for Chinese^2^), but significantly higher specificity. The terms “sensitivity” and “specificity” here relate to the accuracy of the tool. A tool’s sensitivity shows how many positive cases are detected out of the total pool of positive cases, expressed as a percentage score. The specificity score refers to how often a tool accurately identifies a patient as having a negative result — e.g. the tool detects no diabetic retinopathy where there indeed is no diabetic retinopathy. In diagnostic terms, a low sensitivity score would mean a high chance of a false negative, and a low specificity score a high chance of a false positive.

Compared to unaided clinical judgment (99%), the DLSDR system was lower in specificity across all ethnic groups: 82% for Malay; 73% for Indian, and 76% for Chinese patients.^3^ DLSDR therefore had the highest specificity for Malays (Ting et al. 2017, 2217–2220). DLSDR had the lowest sensitivity for Malay patients: 97% for Malays, 99.3% for Indians, and 100% for Chinese. Malay patients were the most likely to be underdiagnosed. Used as Ting et al. (2017) designed it, the DLSDR performs the initial grading of the retinal images, a curated sample (with a 10% false negative rate) is then passed on to human graders for review. Since DLSDR and the human graders have roughly equal sensitivity, but human graders have higher specificity, this process ought to streamline workflow and improve the overall accuracy and efficiency of human graders.

## Social Context

Singapore is a multi-ethnic, multicultural and multilingual society, and has been since its founding as a city in 1819. As of 2020, according to the Singapore Department of Statistics, Singapore’s ethnic makeup was nearly three-quarters Chinese (74.3%), 13.5% Malay, 9% Indian, and 3.2% other. Historically, the Malays have the longest history in the region. Yet, like much of Southeast Asia, Singapore has been a hub for trade and migration of people for centuries. While the British colonial period (roughly 1819–1963) saw waves of both Chinese and Indian migration, the Chinese population has connections to Singapore that date back at least 700 years, and the long history of Indian influence in the region can be seen through the number of loan words within the Malay language that persists today (Chong and Bak 2019; Low 2004).

In Singapore, Malay patients are currently less likely to be referred to specialists for diabetic retinopathy than non-Malays. Malays account for at least 20% (according to Singhealth 2017) of diabetic patients in Singapore and are therefore at higher risk for developing diabetic retinopathy than non-Malays, especially given that they also experience poorer disease control (due at least in part to the lower engagement in health-seeking behaviours among Malays), which makes it more likely that they will experience this and other complications of unmanaged diabetes. In an unbiased system, we would expect that rates of referable diabetic retinopathy to roughly track the proportion of diabetics who are Malay (> 20%) weighted by the average degree of disease control. Despite this, they are still referred to specialists less often than other groups. Only 7.3% of those referred to specialist care are Malay (Sia et al. 2020).^4^ This would seem to indicate some background bias or other prior unjust disparities present in the social environment in which the DLSDR system would be deployed. We must ask, what explains this disparity? How we account for the unexpectedly low rates of referral (7.3%) in the clinical setting prior to the DLSDR system’s use will influence the permissibility of introducing an AI tool that performs differently for different ethnic groups.

In theory, the background bias in referrals could derive from multiple sources: including personal (conscious or unconscious) bias on the part of physicians or graders; disparities in patient participation in screening; distrust of the medical establishment; workplace discrimination; poverty; religious fatalism; lack of education; cultural norms or personal choice for example (see Kaur-Gill 2022; Zainal et al. 2021; Mohammad 2021). A study by Huang et al. (2009) found that Malays in Singapore were more likely to be unaware that they had diabetes or diabetic retinopathy. This tendency itself could be also attributable to the lower consumption rate of preventative care among Malays when compared with non-Malays in Singapore (Ministry of Health 2020). This lack of uptake of preventive care may itself indicate a dearth of trust among the Malay population, or a lack of opportunity to seek such care due to other socio-economic constraints, like working in the gig economy (Mohammad 2021).

This economic reality places an additional barrier to participating in health screening, since they are unlikely to have the luxury of paid leave. Since Malays, on average live closer to the poverty line (see Sulaiman 2022), the urgency of that loss of income would be more severe, again feeding into a reticence to take time off of work for medical screening. It has been shown that Malays experience workplace discrimination, as the ethnic Chinese majority in Singapore has been found, on average, to be inclined to judge Malay candidates as being less competent than non-Malay candidates despite being similarly credentialled (Chew et al. 2019).^5^ As a result, Malays might reasonably perceive that frequent work absences would feed into the negative assumptions about them, and hence put their job at risk. This, again, could easily translate into a reticence to take time off from work for medical check ups.

The potential for prejudice on the part of clinicians, whether conscious or not, is also important to consider. This would naturally feed into another barrier to participation in screening in the form of distrust towards the medical profession (Goh et al. 2022). This has been found, in other countries, to be causally linked to prior injustices (Best et al. 2021; Wiencek 2023). A recent report by the Community Action Network (Singapore) (n.d.) to the UN Committee on the Elimination of Racial Discrimination on Singapore’s Compliance with the International Convention on the Elimination of All Forms of Racial Discrimination in Singapore has observed that ethnic minorities (Malays and Indians) experience a higher incidence of chronic illnesses as well as higher mortality rates compared to ethnic Chinese, “indicating systemic inequalities in healthcare … that occur along ethnic lines, suggesting that there is indeed racial discrimination in the right to public health”. The lower referral rate could also be spurred on by conscious or unconscious racial bias on the part of clinicians (Wong 2021). It is possible that family physicians might be less willing to send Malay patients for screening or less willing to refer them to specialists despite the grader positively identifying their scans as displaying referable diabetic retinopathy. Indeed, it has been found that the most commonly cited reason that patients do not engage with the health system is prior bad experiences, including experience of discrimination, judgemental or dismissive treatment, and lack of therapeutic rapport or respect (Taber et al. 2015).

Language barriers could also be an issue, as it has been observed in other contexts that language barriers can play a large role in a patient’s likelihood of accessing screening and treatment (Zheng et al. 2012). In Singapore, there are four national languages (English, Malay, Mandarin, and Tamil), and while the modern healthcare system is conducted predominantly in English, many of the older generations of Singapore are likely to be more comfortable in another language. Again, the Community Action Network (n.d.) report describes, “Despite being two of the four official languages in Singapore, there is a lack of readily available Malay and Tamil language interpreters in healthcare settings”. Interestingly, in these reports of health discrimination, the group most commonly reported as experiencing discrimination within the Singapore health system is those of Indian ethnicity. If health provider discrimination were the sole cause of the disparity in diabetic referrals, we would expect to see a similar gap for the Indian community as observed for Malay patients.

A study by Lim et al. (2013) found that in Singapore’s rapid modernization between 1965 and 2009, life expectancy increased across all groups. Additionally, though there was a “convergence in life expectancy between Indians and Chinese, the (substantial) gap between Malays and the other two ethnic groups has remained (Lim et al. 2013). This data, together, would suggest that while discrimination is very likely a component of the explanation for the disparity in diabetic retinopathy referrals experienced by Malays, it is also likely that other factors beyond systemic discrimination in the health system are at play.

Other factors, including some specific to the Malay-Muslim community in Singapore, play a role in shaping both the disease risk, level of control, and likelihood of referral. On one hand, Malay-Muslim people may be motivated to maintain their health as an important part of the religion (part of “good stewardship” of the body, given to them by God) (Zainal et al. 2021). On the other hand, religiously-inspired fatalism, whereby someone may be unwilling to seek medical attention because they regard whether one lives or dies as up to God’s (Allah’s) will, is also common (Goh et al. 2022). Additionally, as Zainal et al. (2021) describe, Islamic norms of modesty (which emphasize the need for women to cover all parts of the body, except for hands and face) as well as traditional gender roles can constrain Malay-Muslim women’s ability to engage in physical exercise. This is a practical constraint on their ability to engage in healthy behaviours, as well as a possible barrier to seeking health care and physical exams more broadly.

Even the prevalence and distribution of diseases themselves are not immune to social pressures. As we can see with a condition like diabetes, the risk factors for developing the condition and the ease of managing it are both subject to social conditions. The freedom to take the time to exercise as well as the affordability and availability of healthy food options are not evenly distributed, and these things tend to be harder to acquire for those who are socially disadvantaged (for whatever reason). For Malays in Singapore, then, the fact that they experience both a higher incidence of diabetes and poorer disease control is likely linked.

All of these factors likely intersect to contribute to present health disparities experienced by Malay patients in Singapore. Though an exact diagnosis of the origin of the inequalities experienced by Malays in Singapore is beyond the scope of this paper, what we have presented here gives us reason to believe that at least some non-negligible proportion is insidious in nature. Given that at least some portion of the existing disparities are rooted in inequality and bias, the potential widening of a health disparity is concerning.

Implementing the DLSDR into this social context would likely result in more referrals to specialists across groups when compared with unaided clinical judgment due to the boost in efficiency that the system provides. However, it could also widen the existing disparity between Malays and non-Malays. This would be because while the sensitivity in detecting diabetic retinopathy improves for all groups, the sensitivity improves for non-Malays more than Malays. Meanwhile, most if not all of the causes of the disparity remain untouched by the implementation of the DLSDR.

## Algorithmic *Bias* in an Imperfect World

Bias comes in numerous forms, algorithmic, social, and statistical. Algorithmic bias occurs when the prediction or outputs of a model would, if implemented under prevailing circumstances, unfairly and unjustifiably benefit or disadvantage certain individuals or groups (Kordzadeh and Ghasemaghaei 2022).^6^ There are a variety of different sources of algorithmic bias; we do not explore these in depth because it has been well canvassed elsewhere (see O’Neil 2017; Mittelstadt et al. 2016; Mittelstadt et al. 2023; and Marjanovic et al. 2022). Bias can influence an AI model at various stages, from the way in which the problem is framed, the process of curating and preparing the data, bias being present in the data itself, through to inappropriate deployment in a new context where it has not been validated on local data, or failure to consider the social context in which the model is deployed (Leslie et al. 2021). Training data may be statistically biased because it is unrepresentative of the target patient population. For example, studies into tissue-based functional genomics (investigating the impact of genomic diversity on human biological function including health and disease) currently rely on samples from donors who are predominantly of European descent (ranging from 82 to 95%) (Long et al. 2022). Research populations often fail to reflect patient populations in terms of age, gender, race, socioeconomic status, comorbidities, and disease severity (Mosenifar 2007). Real-world data captured by electronic health records is more representative than research data but still fails to account for the unmet health needs of patients who cannot or do not (for various reasons) access health care when sick. To minimise statistical bias, AI algorithms should be trained with large datasets of diverse and balanced information (Agostina et al. 2020).

Alternatively, bias in data used to train the AI program may be due to systemic social bias such as differential access to the underlying social determinants of health (e.g. experiences of poverty, lack of education and living conditions); differential access to healthcare (e.g. distance to hospital or insurance status); or patterns of discrimination (such as racism and sexism). Hence, even when the data used to train the model may be representative and accurate, it may still capture and reflect objectionable aspects of the real world such as stigma, discrimination, or oppression (Mitchell et al. 2021). For example, an unconscious racial bias that leads to the over-policing of people of colour in the USA can generate statistical bias in arrest data if people of colour are more likely to be arrested and if arrests are considered a proxy measure of actual crime (Lum and Isaac 2016). If we were, hypothetically, able to find a perfect measure of the crime rate this would be free from statistical bias but still be coloured by social bias. This is because individuals’ participation in crime is in part socially influenced and reflects unequal social structures, and societal bias arises even in how crime is defined (Raphling 2018). There is no neutral health data set that does not include these biases and therefore the impact of social bias on AI programs is impossible to avoid.

Thus, it seems to be the case that no AI can be made completely unbiased. Likewise, it is also true that no social context in which a given AI might be employed is completely unbiased. Thus, we will begin our arguments from a position we think is most reasonable: It is unclear what proportion of the inequalities have blameworthy roots, but it is a near certainty that some undefined, but non-negligible proportion is.

We have stipulated that both utility and equity are important considerations for any healthcare policy. Bias exists not just in the algorithms that we create, but in the environments where such tools would be deployed as well. It seems at present that we are unable to completely remove bias or inequality from either. This forces us to consider the following questions: When might it be permissible to use a biased AI in an already biased world? And is it ever permissible to worsen an existing disparity for the sake of utility?

## The Impact of *Bias* on Utility and Equity

Correcting for fairness within the AI model is notoriously difficult and requires differentiating and balancing bias, variance, and noise (Petersen et al. 2023). While many data scientists are working to improve statistical metrics for fairness, we approach this case study from the perspective that most AI training data in health is biased (both socially and statistically) to some extent, and even if app developers have an ethical obligation to reduce bias where possible, we must expect that some residual bias will remain. The purpose of this paper is to consider the ethical trade-offs between equity and utility in the context of a specific case study. The bottom-up, case-based approach we take here provides a useful complement to more abstract discussion of algorithmic fairness and shows how consideration of bias might play out in a clinical context. In our view, judgments about an acceptable degree of bias and how this should be weighed against health utility must account for normative, political, and social aspects of the specific context, health condition, and AI tool. This requires consideration of the social, economic, and historical context and current barriers and enablers to healthcare access.

When thinking about AI in the context of health we might find that utility and equity intersect in the following ways, wherein the AI model could:

- Be less biased than current clinical care models, resulting in decreased health disparities between groups
- Be as biased as current clinical care models, but offering improved aggregate population health utility
- Be more biased than current clinical care models, resulting in increased health disparities between groups, but offering improved aggregate population health utility *and* improves the health of the disadvantaged group

In order to make the comparison with current clinical models viable, we might think of an AI as being more biased if it would create new or exacerbate existing unjust disparities in society (Panch et al. 2019). If an AI is more biased than current clinical care models, then there is a strong, if not wholly decisive, reason to not implement the AI. The DLSDR case that we present in this paper, seems exceptional in that the AI is more biased than current clinical models and yet there is no strong reason against implementing it.

In part, this is due to the nature of the particular forms of bias at play in this case. As mentioned, it seems reasonable to conclude that at least some of the existing background disparity in Malay referrals was rooted in blameworthy forms of bias. It is also true that although the DLSDR system was slightly more biased than the status quo, nevertheless the quality of that bias is likely to be less objectionable.

We know that DLSDR is a biased system, in that it is more likely to underdiagnose Malays than it is to underdiagnose other groups, while at the same time, it is also more biased than the status quo. The question of the ethical permissibility of using such a biased system depends on its interactions with the present social context, which we also know to be characterised by bias and inequality. How these different layers of bias interact is determined at least in part by the quality of the background bias (as discussed in section II). We postulate that it is permissible to implement a biased AI tool where (1) its introduction reduces the influence of biases, and/or (2) where the utility gained is significant enough and shared across groups. We know that algorithmic bias in this case is likely to contain some social bias, though how much is unknown. In what follows, we will explore some possible sources of the background inequality experienced by Malays in this case, and how this may shape our assessment of the permissibility of the AI tool.

## The Question of Permissibility

How does the diagnosis of the origin of the disparities between Malays and other races in terms of diabetic screening and referrals affect the permissibility of the DLSDR system in this case?

If grader bias is the origin of most of the disparity, employing the AI will almost certainly reduce this disparity, since it would remove such biased individuals from the process. Grader bias occurs if the trained graders are more likely to underdiagnose Malay patients than non-Malay patients. To see why the disparity would be reduced, let us consider the hypothetical situation where the disparity in referrals is explained entirely by grader bias. This means that Malay patients attend screening at roughly the same rates as their non-Malay counterparts. In such a situation, the percentage of attendees who are Malay should reflect the percentage of diabetics who are Malay or exceed it. Of those who attend the screening, the percentage of Malay patients with referable diabetic retinopathy who are actually referred would be about 33%, and the percentage of non-Malay patients with referable diabetic retinopathy would have to be above 90% (Ting et al. 2017; Sia et al. 2020). That is the only way a mere 7% of those who are referred could be Malay if the percentage who attend screening is about 20%. By contrast, given the same rate of screening attendance, with DLSDR, the percentage of Malay patients with referable diabetic retinopathy who are referred would be 97%, while for non-Malays, it would be at 100% or very close. Of course, these numbers only describe a scenario wherein diabetic Malays seek screening at the same rate as diabetic non-Malays. Moreover, this situation may be very unlikely.

While some have argued that humans are often more prone to bias than algorithms (Kleinberg et al. 2019; Kahneman et al. 2021; Sunstein 2021) due to cognitive bias, it is unclear which cognitive bias is operating on graders such that they are somehow more likely to under-diagnose anonymised retinal scans from Malays than from non-Malays. To be sure, there are interethnic differences in the fundus pigmentation of eyes with Indian and African populations having darker pigmentation than Chinese and White populations (Cheung et al. 2007; Rochtchina et al. 2008; Ting et al. 2017). However, while differences in fundus pigmentation have been hypothesised to affect the measurement of retinal vessel calibre, there is no evidence to suggest that it affects the measurement and detection of retinopathy. Given the other possible factors outside of grader bias that prevent Malays from seeking screening, the real-world difference will likely be less dramatic or likely even biased in the other direction. Nevertheless, if grader bias is indeed a cause of the disparity, implementing DLSDR would result in a potentially significant reduction in that disparity and would make the AI preferable to unaided decision-making in this case.^7^ By taking the biased graders out of the loop and replacing them with a biased AI screening tool, both Malays and non-Malays stand to benefit.

If all, or almost all the disparity is caused by other factors which reduce rates of participation in screening (like religious fatalism, socio-economic disparities, suspicion of the medical system, or biased family physicians being less likely to send Malays for screening) or otherwise contribute to reduced rates of referral (like a biased physician being less likely to refer Malay patients to specialists despite positive diagnosis by graders), then the disparity will be likely to increase when DLSDR is implemented. This is because these causes of the disparity are not removed when the AI is introduced. Meanwhile, the AI introduces a new source of inequality. To see why, consider the case where the differential referral rate is due entirely to lower rates of participation in screening. If the AI is implemented, we might expect the proportion of Malays referred for diabetic retinopathy to drop even lower to 7.1%^8^ (Sia et al. 2020). While this drop of 0.2% might not seem like much, it does count as a widening of the disparity (see Appendix for a walkthrough of the math).

In this way, it can be seen that a diagnosis of the origin of the background inequalities and the nature of the existing bias in the social context has implications for the acceptability of implementing a biased AI system. Given the details of the case, it seems that implementing DLSDR is likely to improve the referral rates for Malays if the original disparity was explained by mostly unjust forms of bias such as clinical bias in referral rates. This is true even if the DLSDR system itself is not entirely free of bias. Conversely, the more neutral the origin of the existing disparity is — for example, religious fatalism as a cultural barrier to screening — the less likely the DLSDR system is to improve upon the disparity — and in fact, may widen the gap because the AI toll will increase detection rates and referrals for specialist care for other ethnicities but not for Malay patients.

Let us turn our attention back to the central tension in this case: tradeoffs between utility and equity. The mere fact that an AI intervention widens pre-existing disparities may not be enough to show that using it is impermissible. Before we can make a ruling one way or another, we must explore the utility that stands to be gained.

As mentioned in the case study, the DLSDR system is comparable in accuracy compared to human experts. Implementing it as it was designed should have the effect of making screening for diabetic retinopathy cheaper and quicker, which has the potential to translate into serving more people and catching and treating more cases. For the average Singaporean who might otherwise be affected, having the condition caught early can mean avoiding blindness, job loss, and other consequences. To suggest that we withhold what would be a significant benefit for the majority of the population because it did not manage to do so without worsening a disparity is to weigh equity higher than utility. Doing so in this case seems counterintuitive for a number of reasons.

One significant consideration in favour of implementing DLSDR is that despite the gap between Malays and non-Malays increasing, in absolute terms, Malays will still be better off on average with the DLSDR tool than without it. After all, while a human grader’s sensitivity can go as low as 90%, the AI tool’s sensitivity for Malays is 97% (Ting et al. 2017; Sia et al. 2020). Of the patients who attend screening and have diabetic retinopathy, 7% more will be referred to the specialist when using the AI than without the AI. As a result, more Malay patients will have access to specialist care, and thereby be better enabled to manage their condition. Though this gain comes only by means of a trade (sacrificing some degree of equity), it may translate into real benefits in wellbeing, as well as wider social benefits, through the prevention of job loss and disability. So, with a full accounting of the social context, the trade seems a fair one to make, even if it is not perfect. Ideally, we would like to eliminate disparities and raise everyone’s standard of wellbeing equally. In the absence of that option, however, this seems to be a worthwhile outcome.

Moreover, the difference in sensitivity, although statistically significant, is small: 97% vs 99 or 100%. While we should aim at equity by bringing up the sensitivity for Malays, we should not delay the roll out of such AI which stands to benefit all significantly. The amount of inequity matters, and in this case it is small.

That said, the exact degree of utility created depends on the manner in which the technology is implemented in the clinical context. As discussed, it is likely that at least some of the disparity in referrals is rooted in differences in participation in screening across groups. The DLSDR system does not by itself reduce any burden on the patients being screened, but if it were to be rolled out in a manner that did so, then the disparity would naturally shrink. Thus, we would recommend that DLSDR be implemented in partnership with efforts to maximise screening,^9^ for example by ensuring that screening is delivered in a way that maximises accessibility and cultural acceptability for Malay patients. As the absolute number of Malay diabetics getting screened increases, more would benefit, despite the algorithm’s internal bias.

In this analysis, it can be seen that implementing an imperfect system like DLSDR has the potential to meaningfully improve the referral and treatment of diabetic retinopathy for the Malay population, even though, in a worse-case scenario it could widen the gap in referral rates thereby increasing a health disparity between ethnic groups. Implementing the tool has the potential to mitigate differential referral rates based on conscious or unconscious racial bias on the part of clinicians, and also increase the absolute number of Malays that are diagnosed and referred. In a situation like the DLSDR case study, as with many, there is no perfect outcome. Rather, we are presented with a choice between two types of trade-off. In the case that we implement the AI app, we worsen some inequity in exchange for a meaningful amount of utility, and that utility is shared among all ethnic groups in Singapore (even if unevenly). In the case that we do not implement the DLSDR, then we maintain the current level of inequity and miss out on the utility that could have been gained. This opportunity cost seems tough to justify. Thus, implementing the AI here is the preferable option.

The DLSDR case study is an example of an AI system with relatively low bias, and we have argued that because of the particulars of this case, it is permissible to implement the system regardless of the fact that a pre-existing disparity is worsened. Additionally, due to the particulars of this scenario, though the disparity was increased, the Malay population still benefits in the absolute sense.

In a hypothetical alternative scenario — wherein Malays do not enjoy any of the benefits of this system and the utility gained is only enjoyed by the majority group —, there would be less weight on the scale in favour of permitting the algorithm’s use. While we contend that it is permissible to widen the inequality in the DLSDR case, at least part of the reasoning is rooted in the fact that all boats rise. Thus, if there was no benefit to be found for the Malay population (in either the relative or absolute sense) then the use of such an algorithm would be less justifiable. However, if additional measures were taken (like some kind of compensation or offset being given to the disadvantaged group), it could still be permissible to employ the biased system, if the gain in utility is considered important enough.

Before we move on to the conclusion, it is worth entertaining one final concern. One possible remaining objection to employing a biased system like this (even when the disparity is only slightly worsened, and even when utility is gained for all groups) could still be raised on the grounds that its use expresses some kind of disrespect for members of the disadvantaged group. If employing a biased algorithm that worsens a pre-existing unjust disparity is considered disrespectful or disparaging, then we have at least one more reason not to condone its use (see Lippert-Rasmussen (2023) for more on this). While we agree in the abstract that this is a valid concern, in the case of DLSDR, it is difficult to see how its use would be disrespectful to the Malay population, given that it is still likely to increase the total number of diabetic retinopathy patients being diagnosed. Additionally, it would seem to matter what the origin of the lower accuracy in the minority group is rooted before one can claim that an algorithm’s use is disrespectful. Many medical devices fail to be equally accurate across all possible phenotypes (for example, pulse oximeters are often less accurate on darker skin). If the bias of the DLSDR system was rooted in a similar origin, it is hard to see how this would be related to disrespect. Further, if one does take the use of such a device to be disrespectful, then many common medical devices will be just as guilty (e.g. the pulse oximeter).

## Conclusion

When faced with the need to choose between competing goods like utility and equity, top-down reasoning and generalizations are not always sufficient. In an ideal world, of course, we would never need to trade between the two. However, we do not live in an ideal world, and in practice, trade-offs are often unavoidable. The nature and extent of inequality and utility matter in weighing these two values. In this paper, we have shown that a bottom-up, case-based approach can illuminate important nuances in the question of how to navigate some of the ethical dilemmas presented by the use of biased AI in healthcare. The case of the AI screening tool for diabetic retinopathy represents one instance where the gains in utility justify the clinical deployment of the tool, despite the potential equity implications. Through our exploration of this case, we hope to have offered some useful lessons for when an AI ought to be implemented even when doing so may create or widen disparities, even ones that are admittedly unjust. Our analysis of the ethical trade-offs involved in this case is context-specific — grounded in the nature of the social, cultural, and historical contacts within Singapore. The lesson from the current case seems to be that pursuing utility at the expense of equity is at least sometimes permissible, as long as that gain in utility is enjoyed by all groups involved. We should not let the permissibility of implementing the AI tool in this case prevent us from trying to develop less biased versions of the AI going forward. Nevertheless, until the less biased version is available, it is appropriate to implement the imperfect one. Waiting for perfection would mean a loss in well-being born by all members of society. Those who would wait for perfection may indeed be waiting for a long time.

## Appendix

We are assuming here that the disparity in referrals is explained entirely by differences in rates at which Malays and non-Malays attend screening for diabetic retinopathy. For simplicity, we will assume that a patient is referred if and only if the trained grader detects referable diabetic retinopathy. To see why the disparity in referral rates would worsen, consider a hypothetical where there are currently 1000 patients with diabetic retinopathy being referred to the ophthalmologist for said condition.

At current rates of referral,

1000 × 7.3% = 73 of these patients would be Malay and

1000 × 92.7% = 927 would be non-Malay.

At current unaided sensitivity rates of about 90% (Ting et al. 2017; Sia et al. 2020), the number of Malay patients who had diabetic retinopathy and attended screening would be

100 × 73/90 ≈ 81

Likewise, the number of non-Malay patients who had diabetic retinopathy and attended screening would be

100 × 927/90 = 1030

Under the algorithm, the sensitivity for detecting diabetic retinopathy among Malays would be 97%. As such, the number of Malays who are detected and referred would be

0.97 × 81 = 78.6 ≈ 79

Likewise, given that the algorithm has 100% sensitivity for non-Malays, the number of non-Malays who would be referred is 1030.

With the AI, the percentage of referrals who would be Malay is:

100% × 79/(1030 + 79) = 7.1%

There would thus be a 0.2% drop in the Malay share of referrals even if more Malays were referred to specialists for diabetic retinopathy.

We might try to weaken the assumption that a patient is referred to if and only if their scan is graded as displaying referable diabetic retinopathy. Suppose, instead, that a patient was not always referred to a specialist when the grader detected referable diabetic retinopathy. This might be because the question as to whether a patient should be referred is not settled by the scan result. Suppose only a fraction, q, of non-Malay patients who are graded as having referrable DR are in fact referred. Suppose further that as a result of physician bias an even lower fraction, r of Malay patients who are graded as having referrable DR are referred to a specialist.

Consider, again, a hypothetical where there are currently 1,000 patients with diabetic retinopathy being referred to the ophthalmologist for said condition.

At current rates of referral,

1000 × 7.3% = 73 of these patients would be Malay and

1000 × 92.7% = 927 would be non-Malay.

At current unaided sensitivity rates of about 90% (Ting et al. 2017; Sia et al. 2020), the number of Malay patients who had diabetic retinopathy and attended screening would be

100 × 73/90r ≈ 81/r

Likewise, the number of non-Malay patients who had referable diabetic retinopathy and attended screening would be

100 × 927/90q = 1030/q

Under the algorithm, the sensitivity for detecting diabetic retinopathy among Malays would be 97%. As such, the number of Malays who are detected would be

0.97 × 81/r = 78.6/r ≈ 79/r

Since the physician is still in the loop and has to refer the patient to the specialist, the number of Malays who would be referred is

79/r × r = 79

Likewise, given that the algorithm has 100% sensitivity for non-Malays, the number of non-Malays who would be detected is 1030/q

The number of non-Malays who would be referred is

1030/q × q = 1030

With the AI, the percentage of referrals who would be Malay is:

100% × 79/(1030 + 79) = 7.1%

There would thus still be a 0.2% drop in the Malay share of referrals even if the disparity is partly contributed to by biased physicians being more reluctant to refer Malay patients despite the patient’s scans being graded as presenting with referrable DR.

## Footnotes

1 Using the results provided by Ting et al. (2017), we reconstructed the number of true positives, false negatives, false positives, and true negatives for each ethnic group. We then conducted a Fisher’s exact test using these numbers. Comparing sensitivity of Malays vs. Indians and then vs Chinese, the *p*-value was at 0.0021 and 0.001 respectively, which is well below 5% significance. By comparison, when comparing the difference in sensitivity between Indian and Chinese, the *p*-value was 0.3497 which indicates there is no significant difference between those groups.

2 These numbers reflect the scores when the DLSDR system is set to maximise sensitivity. This setting can be adjusted, but it was determined by the developers that greater sensitivity was preferable to aid workflow of the human graders (DLSDR is intended as a first line screening to narrow the pool of scans for human graders to examine). It is important to note that though this score can be adjusted, perfect parity across groups was not achievable.

3 Figures taken from Table 5 in Ting et al. (2017), page 2220. See also Fig. 2, Graph A in Ting et al. (2017), wherein it can be seen that for those under 60, the specificity can be boosted to 95% (if the graph is accurate). This would be a less significant drop in specificity.

4 For comparison, in Singapore, the proportion of diabetics who are Chinese is 66.2%, while their referral rate for diabetic retinopathy is 78.6% (higher than would be expected), and for Indians, who represent 13.6% of diabetics in Singapore, their referral rate is 11.7% (Sia et al. 2020). Indians are the least likely to be underdiagnosed, and Malays the most likely to be.

5 see Baker (2019) and Chia (2019) for other examples of discrimination faced by Malays

6 See also Panch et al. (2019); Awan (2023); Barocas and Selbst (2016) for other similarly normatively loaded accounts of bias. Non-normative accounts of bias like Johnson’s (2021) are not in accord with ordinary usage and are thus not able to ground normative inferences.

7 Even if we attributed the disparity in referrals to bias in the referring physician, using the algorithm would not reduce the disparity. Referring physician bias can be conceived of as occurring when physicians are more likely to discount a positive diagnosis by the grader/AI for Malays than for non-Malays. However, replacing the grader with an AI does not eliminate any bias by the physician (see the Appendix for the detailed argument).

8 This is assuming that using the AI does not result in a disproportionately higher rate of participation in screening for Malay patients. After all, if adoption of AI did increase Malay participation in screening significantly more than it did for non-Malay patients, the gap in referral rates could easily narrow. However, there is little reason to think that this would be the case. The AI at best replaces the grader who is downstream from all the factors which reduce participation in screening.

9 This is no small task, however, since screening is still likely to eat up half of a patient’s work day at the least, given that the medication required to dilate the pupils so that images of the retina can be taken impairs vision for four hours after the retina is imaged (and may also cause headaches).
